# Supplementary material for: Content-rich biological network constructed by mining PubMed abstracts
Source: BMC Bioinformatics. 2004 Oct 8;5:147. doi: 10.1186/1471-2105-5-147 (PMC528731; doi:10.1186/1471-2105-5-147)
Supplement: Additional File 5 — The original Chilibot query results of the term "long-term potentiation (LTP)" and 22 other terms, limiting the latest references analyzed to the years 1990, 1995, 2000, and 2004. [file 1471-2105-5-147-S5.bz2 › chilibotAdditionalFile5/ltp2000/html/SYNAPSIN I_AMPA.html]

 


 **SYNAPSIN I** and **AMPA** 
  
Found 4 abstracts in PubMed,  **4 abstracts were retrieved and analyzed**.  


---

 Search Google  |
 PDF files only 
|  EDU domain only 

---

**Interactive relationship** (e.g. stimulation, inhibition, etc)

**Parallel relationship** (e.g. studied together, co-existance, homology, etc.)

- These results support the idea that CaM kinase II contributes to the induction of hippocampal LTP in both postsynaptic and presynaptic regions through phosphorylation of target proteins such as the  **AMPA**  receptor, MAP2 and  **synapsin I** .  Ref: 8740440 Neurochem Int, 1996
- However, exposure of low glutamine cultures to 100 mM ethanol for 4 days significantly increased the levels of NMDA receptor subunits and  **AMPA**  receptor subunits, but had no effect upon kainate receptor subunits or the synapse associated proteins  **synapsin I**  and PSD 95.  Ref: 10069569 Alcohol Clin Exp Res, 1999
- NMDA and  **AMPA**  kainate glutamate receptors modulate dentate neurogenesis and CA3  **synapsin I**  in normal and ischemic hippocampus.  Ref: 11129783 J Cereb Blood Flow Metab, 2000
- The effect of N methyl D aspartate NMDA and 2 aminomethyl phenylacetic acid kainate  **AMPA**  kainate glutamate receptors on dentate cell proliferation and hippocampal  **synapsin I**  induction was examined after global ischemia.  Ref: 11129783 J Cereb Blood Flow Metab, 2000
